# Supplementary material for: Identification of photoperiod-induced specific miRNAs in the adrenal glands of Sunite sheep (Ovis aries)
Source: Front Vet Sci. 2022 Jul 22;9:888207. doi: 10.3389/fvets.2022.888207 (PMC9354845; doi:10.3389/fvets.2022.888207)
Supplement: Supplementary file 1 [file Data_Sheet_1.ZIP › Supplementary file/miRDeep2 file.zip!/miRDeep2 file/Know_Structure/LP42A1/oar-mir-23b.pdf]

miRBase precursor : oar-mir-23b
Total read count : 21387
oar-miR-23b read count : 21355
remaining reads : 32

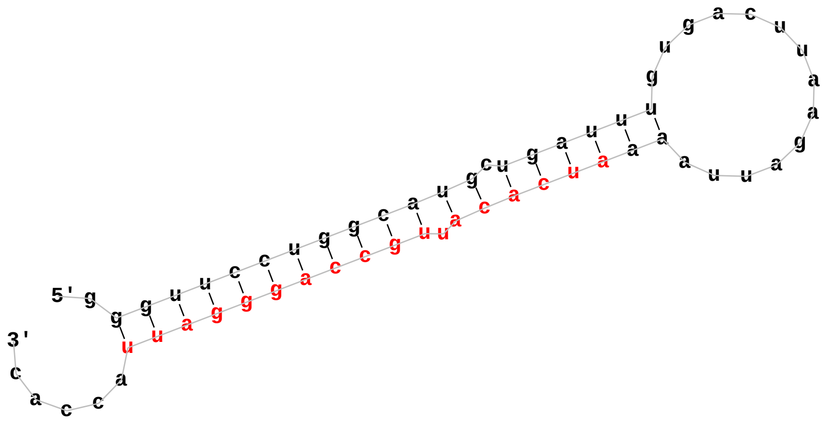

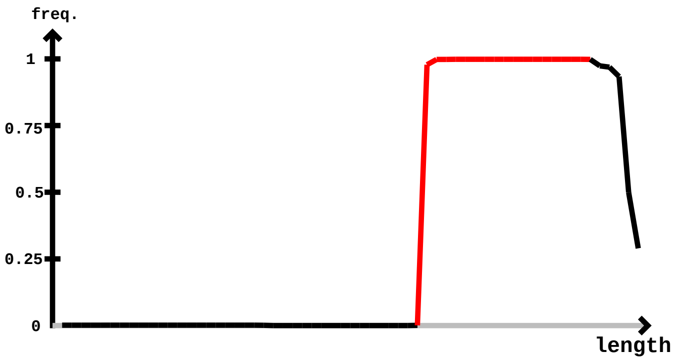

oar-miR-23b

5'- ggguuccuggcaugcugauuugugacuuaagauuaaaaucacauugccagggauuaccac -3'	exp		
((((((((((((((.((((((..............)))))))).))))))))))..))..	reads	mm	sample
ggguuccuggcaugcugauu..	10	0	seq
ggguuccuggcaugcugauuu.......................................	21	0	seq
....uccuggcaugcugauuugugacu.................................	1	0	seq
....................................aaucacauugccagggau......	1	0	seq
....................................aaucacauugccagggauu.....	1	0	seq
....................................aaucacauugccagggauuac...	1	0	seq
....................................aaucacauugccagggauuacc..	10	0	seq
....................................aaucacauugccagggauuacca.	2	0	seq
....................................aaucacauugccagggauuaccac	2	0	seq
.....................................aucacauugccagggaC......	1	1	seq
.....................................aucacauugccagggau......	5	0	seq
.....................................aucacauugcUagggau......	1	1	seq
.....................................aucacauugccUgggauu.....	2	1	seq
.....................................aucacauugccagggauu.....	511	0	seq
.....................................aucacauugcUagggauu.....	2	1	seq
.....................................aucacauugccagggauua....	103	0	seq
.....................................aucacauugccagggauuac...	736	0	seq
.....................................aucacauugccagggauuacc..	9148	0	seq
.....................................aucacauugccagggauuacca.	4397	0	seq
.....................................aucacauugccagggauuaccac	5995	0	seq
......................................ucacauugccagggauu.....	4	0	seq
......................................ucacauugccagggauuac...	18	0	seq
......................................ucacauugccagggauuacc..	125	0	seq
......................................ucacauugccagggauuacca.	82	0	seq
......................................ucacauugccagggauuaccac	195	0	seq
....cacauugccagggauuacca.	1	0	seq
....cacauugccagggauuaccac	1	0	seq
....acauugccagggauuacc..	9	0	seq
....acauugccagggauuacca.	1	0	seq
....acauugccagggauuaccac	1	0	seq
